# West Nile Virus Infection in the Central Nervous System

**DOI:** 10.12688/f1000research.7404.1

**Published:** 2016-01-26

**Authors:** Evandro R. Winkelmann, Huanle Luo, Tian Wang

**Affiliations:** 1 Department of Microbiology & Immunology, University of Texas Medical Branch, Galveston, Texas, 77555, USA; 2 Department of Pathology, University of Texas Medical Branch, Galveston, Texas, 77555, USA

**Keywords:** West Nile Virus, west nile virus infection in central nervous system, central nervous system infection, flavivirus, arboviral encephalitis

## Abstract

West Nile virus (WNV), a neurotropic single-stranded flavivirus has been the leading cause of arboviral encephalitis worldwide.  Up to 50% of WNV convalescent patients in the United States were reported to have long-term neurological sequelae.  Neither antiviral drugs nor vaccines are available for humans.  Animal models have been used to investigate WNV pathogenesis and host immune response in humans.  In this review, we will discuss recent findings from studies in animal models of WNV infection, and provide new insights on WNV pathogenesis and WNV-induced host immunity in the central nervous system.

## Introduction

West Nile virus (WNV), a mosquito-borne, single-stranded, positive-sense flavivirus, has been the leading cause of viral encephalitis in the US for more than a decade. The virus was originally isolated from Uganda in 1937, later caused epidemic outbreaks in Asia, Europe, and Australia, and was introduced into the US in 1999 ^1^. Human infection results mostly from mosquito bites, blood transfusion, organ transplantation, or occupational exposure ^2, 3^. In addition, WNV transmission through breastfeeding and *in utero* exposure is possible, although recent evidence suggests that the risk is very low ^4– 7^. About 80% of human infections with WNV are asymptomatic. The features of acute illness range from West Nile fever, to neuroinvasive conditions, including meningitis, encephalitis, acute flaccid paralysis, and death ^8^. Up to 50% of convalescent patients with WNV have been reported to have long-term neurological sequelae or develop chronic kidney diseases, or both ^9– 17^. Although serologic and organ screening may reduce the risk of WNV infection through blood transfusion and organ transplantation ^18– 20^, there is no specific therapeutic agent for treatment of WNV infection, and an approved vaccine is not currently available for humans.

Animal models, which recapitulate WNV-induced neurological diseases in humans, have been effective *in vivo* experimental models to investigate WNV pathogenesis and host immune response ^21– 23^. In this review, we discuss recent findings from studies in animal models of WNV infection and provide new insights on WNV pathogenesis and virus-induced host immunity in the central nervous system (CNS).

## West Nile virus entry into the central nervous system

The natural transmission of WNV in humans occurs through mosquito bites ^24^. Keratinocytes and Langerhans cells (LCs) are the initial target cells where the virus is naturally deposited. WNV infection in keratinocytes induces innate cytokine responses mediated by Toll-like receptor (TLR) 7, which further promotes LC migration from the epidermis and accumulation in the local draining lymph nodes, where the virus is amplified before dissemination to kidney, spleen, and other visceral organs ^25– 27^. Following a systemic infection, WNV crosses the blood-brain barrier (BBB) after a brief viremia and ultimately invades the CNS ^28^.

The development of WNV encephalitis is correlated with the ability of the virus to gain access to the CNS (neuroinvasiveness). At present, the mechanisms by which WNV enters the brain are not well understood. As a higher viral burden in serum usually correlates with earlier viral entry into the brain, it has been suggested that WNV infects the CNS in part via hematogenous spread ^29^. The BBB is a complex structure that is composed of the tight endothelium formed by endothelial cells through tight junctions and smooth muscle cells surrounded by a layer of astrocytic foot processes ^30, 31^. Systemic WNV replication-induced innate cytokine responses are known to control BBB integrity ( Table 1). Pro-inflammatory cytokines, including tumor necrosis factor-alpha (TNF-α), interleukin-1 beta (IL-1β), and macrophage migration inhibitory factor (MIF), contribute to the disruption of the BBB ^32– 34^. In addition, matrix metalloproteinase 9 (MMP9), which is upregulated upon WNV infection in both the periphery and mouse brain, facilitates WNV entry into the brain by enhancing BBB permeability ^35^. Semaphorin 7A (Sema7A), a potent stimulator of monocytes and neutrophils, acts upstream of the host inflammatory reaction during WNV infection. Following infection, Sema7A-deficient mice produced less TNF-α in the periphery and had a reduced BBB permeability compared with wild-type controls ^36^. In contrast to the effects of pro-inflammatory cytokines, both type I interferon (IFN) (IFN-α and IFN-β) and type III (IFN-λ) are implicated in promoting BBB integrity. Daniels *et al.* have recently demonstrated that type I IFNs play a direct role in endothelial permeability and tight junction formation via balanced activation of the small guanosine triphosphatases (GTPases) Rac1 and RhoA interactions and indirect suppression of the effects of TNF-α and IL-1β ^33^. The TAM receptors Tyro3, Axl, and Mertk are receptor tyrosine kinases that dampen host innate immune responses upon interactions with their ligands Gas6 and Protein S, which recognize phosphatidylserine on apoptotic cells ^37^. A recent study showed that activation of Mertk synergized with IFN-β to tighten cell junctions and prevent virus transit across brain microvascular endothelial (BMVE) cells. As a consequence, mice lacking Mertk or Axl (or both), but not Tyro3, exhibited greater vulnerability to infection with neuroinvasive WNV ^38^. In another study ^39^, mice lacking IFN-λ signaling were shown to have increased viral titers in the brain and spinal cord during WNV infection. This is not associated with a direct antiviral effect of IFN-λ in the CNS. Instead, IFN-λ signaling in BMVE cells modulates tight junction protein localization in a protein synthesis- and signal transducer and activator of transcription 1 (STAT1)-independent manner, which increases transendothelial electrical resistance and decreases virus movement across the BBB. Besides innate cytokines, upregulation of cell adhesion molecules (CAMs), such as intercellular adhesion molecule-1 (ICAM-1) and vascular cell adhesion molecule-1 (VCAM-1), and E-selectin expression on WNV-infected BMVEs induces the adhesion and transendothelial migration of leukocytes across these cells ^40, 41^ ( Table 1). Moreover, direct infection of BMVE cells facilitates the entry of cell-free virus into the CNS without disturbing the BBB, and an increase on CAM further assists the trafficking of WNV-infected immune cells into the CNS via a ‘Trojan horse’ mechanism, thereby contributing to WNV dissemination into the CNS and its associated pathology ^42– 44^. Finally, infection of the olfactory neurons and consequent dissemination to the olfactory bulb and direct axonal retrograde transport of virus that has infected peripheral neurons have been postulated as the other mechanisms of WNV entry into the CNS ^45– 47^.

**Table 1.** Host factors modulate blood-brain barrier integrity.

Host factors	Effects on BBB integrity	References
Pro-inflammatory cytokines, including TNF-α, IL-1β, and MIF	Disruption of the BBB	32– 34
Matrix metalloproteinase 9	Enhancing BBB permeability	35
Semaphorin 7A	Enhancing BBB permeability by promoting TNF-α production	36
Type I IFNs (IFN-α and IFN-β)	Promoting BBB integrity via balanced activation of the small guanosine triphosphatase Rac1 and RhoA interactions and indirect suppression of the effects of TNF-α and IL-1β	33
TAM receptors Tyro3, Axl, and Mertk	Promote BBB integrity by synergizing type I IFNs	38
IFN-λ	Modulates tight junction protein localization in brain microvascular endothelial cells, which increases transendothelial electrical resistance and decreases virus movement across the BBB	39
CAMs, such as ICAM-1, VCAM-1, and E-selectin	Upregulation of CAM expression induces the adhesion and transendothelial migration of leukocytes across these cells	40, 41

BBB, blood-brain barrier; CAM, Cell adhesion molecules; ICAM-1, intercellular adhesion molecule-1; IFN, interferon; IL-1β, interleukin-1 beta; MIF, macrophage migration inhibitory factor; TNF-α, tumor necrosis factor-alpha; VCAM-1, vascular cell adhesion molecule-1.

The host has developed multiple strategies to limit virus dissemination in the periphery and prevent the trafficking of WNV across the BBB. First, WNV infection activates the signaling pathways of several pattern recognition receptors (PRRs), including TLRs 3 and 7 and RIG-I-like receptor (RLR), in order to boost innate immunity, culminating in the synthesis of antiviral cytokines, such as type I IFNs and pro-inflammatory cytokines ^34, 48– 50^. Type 1 IFNs and IFN-stimulating genes (ISGs) both participate in the control of viral infections and prevent WNV from invading the CNS ^51– 54^. Next, innate immune cells such as γδ T cells (in particular, the Vγ1 ^+^ cell subpopulation) expand significantly during WNV infection and play a role in the early control of WNV dissemination mainly through the secretion of IFN-γ ^55– 57^. These cells are also involved in the regulation of adaptive immunity against WNV infection and persistence, presumably mediated by the crosstalk between γδ T cells and dendritic cells (DCs), either via direct contact or indirectly through secreting molecules, to promote DC maturation and activation and ultimately to help T cell priming during WNV infection ^58^. TCRδ ^–/–^ mice (γδ T cell-deficient mice) were shown to have a numeric and functional reduction in memory T cell responses to WNV infection compared with wild-type mice ^59^. Furthermore, B cells and specific antibodies are critical in the control of disseminated WNV infection but are not sufficient to eliminate it from the host ^29, 60– 62^. In particular, induction of a specific, neutralizing IgM response early during infection limits viremia and dissemination into the CNS and protects the host against lethal infection ^29^. In addition, the complement system has been shown to control WNV infection, in part through its ability to induce a protective antibody response and by priming adaptive immune responses through distinct mechanisms ^63, 64^. αβ T cells ^61^ provide long-lasting protective immunity and contribute to host survival following WNV infection. Among them, CD4 ^+^ T cells respond vigorously in the periphery ^65^, whereas CD8 ^+^ T cell responses have been observed in both the spleen and brain following WNV infection ^66^. CD4 ^+^ T cells provide help for antibody responses and sustain WNV-specific CD8 ^+^ T cell responses in the tissues that enable viral clearance ^67^. CD8 ^+^ T cell responses are critical in clearing WNV infection from tissues and preventing viral persistence ^68^. Furthermore, higher levels of peripheral regulatory T cells (Tregs) after infection are known to protect against severe WNV disease in immunocompetent animals and humans ^69^. Tregs are also critical in generating a pool of WNV-specific memory T cells in the periphery ^70^. RLR-mediated innate signaling is involved in regulating adaptive immunity against pathogenic WNV. Mice deficient in mitochondrial antiviral signaling protein (MAVS), the adaptor protein for RLR signaling, were reported to exhibit lower neutralization ability of WNV-specific antibodies and increased numbers of virus-specific CD8 ^+^ T cells, with non-specific immune cell proliferation in the periphery ^71^.

Aging is a known risk factor for WNV-induced encephalitis ^23, 72, 73^. The decline in immunity seen in the elderly is a significant contributor to the increased risk of infection. In an aged mouse model of WNV infection, Vγ1 ^+^ cells displayed a slower, reduced response to WNV infection compared with those of young adult mice ^57^. Although the young adult CD4 ^+^ or CD8 ^+^ T cells readily protected immunodeficient mice upon WNV infection, T cells of either subset in aged mice were unable to provide WNV-specific protection ^74^. Furthermore, a recent study ^75^ reported that age-dependent cell-intrinsic and environmental defects in the draining lymph nodes result in delayed immune cell recruitment and antigen recognition by CD4 ^+^ T cells, leading to impaired IgM and IgG and increased susceptibility to WNV infection in old mice.

## West Nile virus infection and West Nile virus-induced immune responses in the central nervous system

Once in the brain, WNV can infect and replicate in various types of CNS residential cells, including neurons, astrocytes, and microglial cells ^76, 77^. In a recent study, by using a spinal cord slice culture (SCSC) model, CNS-resident cells were demonstrated to have the capacity to initiate a robust innate immune response against WNV infection in the absence of infiltrating inflammatory cells and systemic immune responses ^76^. CNS cells display differential susceptibility to WNV infection, dependent on their cell-intrinsic host defense programs and cellular activities ( Table 2). The antiviral action of ISG-Ifit2 restricts WNV spread in the CNS, especially during the early stages of virus spread ^78^. Granule cell neurons (GCNs) of the cerebellum, which comprise the largest population of neurons in the brain, express high levels of genes associated with the host defense pathway, including a STAT1- and IFN-dependent signaling signature both at the basal level and after IFN-β treatment. These cells are less susceptible than cortical neurons to WNV replication ^79^. Multiple PRR pathways are involved in the induction of antiviral responses in neurons. IL-1β production triggered by non-obese diabetic (NOD)-like receptor family pyrin domain containing 3 (NLRP3)-inflammasome suppressed WNV replication in neurons ^80^. Both myeloid differentiation primary response gene 88 (MyD88)- and TLR3-mediated innate signaling play protective roles against WNV infection, in part by inhibiting replication in subsets of neurons ^81, 82^. Caspase-12 controls WNV infection in neurons by regulating E3 ubiquitin ligase TRIM25-mediated ubiquitination of RIG-I, which induces high levels of IFN responses ^83^. WNV also triggers production of TNF-α, IL-6, and IFN-β in microglia in a TLR3-dependent manner ^34, 84^. In astrocytes, cellular activities such as inhibiting high levels of furin-like protease activity contribute to a reduced susceptibility to the avirulent WNV-MAD78 infection ^85^. WNV infection in CNS cells also triggers the secretion of chemokines, which further facilitates immune cells to cross the BBB. For example, WNV-specific CXCR3 ^+^CD8 ^+^ T cells preferred to move into the cerebellum, where WNV-infected GCNs expressed a high grade of CXCL10 to clear virus ^86^. Accordingly, the CXCL10-deficient mice had decreased CD8 ^+^ T cell infiltration and increased viral load in the brain ^87^. Likewise, the expression of chemokine receptor CCR5 and its ligand CCL5 was prominently upregulated by WNV infection in the CNS. CCR5 has been implicated for the recruitment of leukocytes (including CD4 ^+^ and CD8 ^+^ T cells, natural killer cells, and macrophages) into the brain. CCR5-deficient mice rapidly succumbed to WNV infection and were not able to clear the virus in the brain during the time course of the disease ^88^.

**Table 2.** Central nervous system cell types and West Nile virus-induced immune responses.

Cells	Immune induction or cellular activities, or both	Effects on host or cells
Neurons	WNV infection triggers IL-1β, IFNs, IFN-stimulating genes, or chemokine production mediated via multiple pathogen recognition receptors, including non-obese diabetic-like receptor family pyrin domain containing 3 (NLRP3)- inflammasome, TLR3, myeloid differentiation primary response gene 88 (MyD88), and Caspase-12-dependent-RIG-I	Restrict WNV infection in the CNS or facilitate immune cells to cross blood-brain barrier and enter the CNS
Microglia	WNV infection triggers TLR3- mediated TNF-α, IL-6, and IFN-β responses	Restrict WNV infection in the CNS
Astrocytes	Inhibit high levels of furin-like protease activity	Restrict avirulent WNV infection in the CNS
Monocyte/Macrophage	Migration into the CNS via CCL2-, CCL7-, TLR7-, or IL-23-dependent pathways	Protective or detrimental?
Neutrophils	Recruitment into the CNS mediated by IL-22 and CXCR2	Control of viral infection at the late stage of infection
CD4 ^+^ T cells	Recruitment into the CNS, producing IFN-γ and IL-2, and regulation by IL-1R-dependent CD11c ^+^ cell response	Directly involved in viral clearance, or by sustaining WNV-specific CD8 ^+^ T-cell response in the CNS, or both
CD8 ^+^ T cells	Recruitment into the CNS; producing IFN-γ, or direct killing of infected cells through secretion of perforin and Fas/Fas ligand, or TNF-related apoptosis-inducing ligand (TRAIL)-dependent pathways; dependent on RIG-I-like receptor (RLR) signaling, and mediated by CXCR4 and IL-1β	Control viral replication upon low-dose WNV challenge and induction of immunopathology in the CNS upon high-dose WNV infection

CNS, central nervous system; IFN, interferon; IL, interleukin; TLR, Toll-like receptor; TNF-α, tumor necrosis factor-alpha; WNV, West Nile virus.

Under normal physiological conditions, the integrity of the BBB restricts the infiltration of leukocytes and maintains the CNS as an immune-specialized compartment. Following systemic WNV infection, immune responses in the CNS are induced by infiltrating inflammatory cells, including microglia/macrophages, neutrophils, and effector CD4 ^+^ and CD8 ^+^ T cells ( Table 2) ^67, 88– 91^, and further infection in the CNS residential cells. Monocyte infiltration into the CNS is the hallmark of WNV encephalitis. After entry into the CNS, they differentiate into macrophages and microglia ^88, 92^. Monocytes are presumably protective against WNV infection, as depletion of these cells increased mortality of mice infected with a neurotropic strain of WNV ^93^. However, inhibition of Ly6C ^hi^ monocyte trafficking into the brain by anti-very late antigen-4 (anti-VLA-4) integrin antibody blockade at the time of observation of the first weight loss and leukocyte influx resulted in long-term survival in mice with lethal encephalitis ^94^. Although CCR2, a receptor expressed on Ly6C ^hi^ inflammatory monocytes, is not directly involved in the recruitment of these cells from blood to the brain, it promotes peripheral monocytosis during WNV infection, which is critical for ultimate accumulation of monocytes in the brain, and host protection ^95^. It was demonstrated that the CCR2 chemokine ligands CCL2 and CCL7 are involved in the monocytosis and monocyte accumulation in the brain. The deficiency of CCL7 in mice leads to increased WNV load in the brain and enhanced mortality, suggesting a critical role of CCL7-mediated immune response in host protection ^96^. Furthermore, TLR7- and IL-23-dependent immune responses were known to mediate CD11b ^+^ macrophage migration into the CNS and protect the host from lethal WNV infection ^49^. Neutrophils are another cell type detected in the CNS, but the role of these cells in WNV infection has yet to be elucidated. Evaluation of cell infiltrate in cerebrospinal fluid from human cases of WNV meningitis and encephalitis showed high counts of neutrophils ^97, 98^. Neutrophils were reported to display dual functions in WNV-infected mice. They could serve as reservoirs for virus replication in the periphery at early stage of infection, but are involved in viral clearance at the late stage of infection in the CNS ^43^. IL-22, a cytokine implicated in the modulation of the chemokine receptor CXCR2, is responsible for the recruitment of neutrophils ^99^. Finally, CD4 ^+^ T cells are required for the clearance of WNV from CNS and therefore mice survival, providing help for antibody responses, and sustaining WNV-specific CD8 ^+^ T cell responses in the CNS that enable viral clearance ^67^. They also have a direct antiviral role in the CNS by producing IFN-γ and IL-2 and have a potential for *in vivo* and *ex vivo* cytotoxicity ^100^. It has been demonstrated that IL-1R1 signaling modulates CD11c ^+^ cell-mediated T cell reactivation and promotes virologic control during WNV infection within the CNS ^101^. CD8 ^+^ T cells have an important function in clearing infection from the CNS and preventing viral persistence upon a low-dose WNV challenge ^68, 102, 103^. Nevertheless, CD8 ^+^ T cells are also involved in promoting CNS pathology following high doses of WNV infection ^104, 105^. Thus, depending on the viral dosage, CD8 ^+^ T cells could be involved in both recovery and immunopathology in WNV infection. The effector function of T cells to control virus replication and eliminate the infection is directed through the production of cytokines, including IFN-γ, or direct killing of infected cells through secretion of perforin and signaling through Fas to -Fas ligand, or TNF-related apoptosis-inducing ligand (TRAIL)-dependent pathways ^68, 102, 103^. RLR-mediated innate signaling was reported to shape optimal CD8 ^+^ T cell activation and subsequent clearance of WNV from the CNS ^106^. CD11b ^+^CD45 ^hi^ infiltrating cells (macrophages) are the primary producers of IL-1β within the CNS. By using an *in vitro* BBB model, Durrant *et al.* have shown that IL-1β promotes CXCR4-mediated CD8 ^+^ T lymphocyte adhesion to BMVE cells. Furthermore, inhibition of CXCR4 promotes T lymphocyte entry into the CNS parenchyma, and this increases viral clearance and ultimately improves survival and reduces viral loads ^107^. Thus, perivascular localization might hinder antiviral immune responses during WNV encephalitis.

## Conclusions

In summary, following systemic WNV infection, immune responses in the periphery help to control virus dissemination, whereas CNS immunity is critical for the clearance of virus. WNV has been the leading cause of viral encephalitis in the US for more than a decade. Current efforts on drug development have been focused mostly on the inhibitors of virus replication. Results from animal studies will provide a better understanding of the mechanisms by which flaviviruses enter the brain and induce lethal encephalitis; they may also lead to the development of new strategies to prevent and treat WNV-induced encephalitis.
